# Added sugars, gut microbiota, and host health

**DOI:** 10.1080/19490976.2025.2592431

**Published:** 2025-12-01

**Authors:** Yanbo Zhang, Ryan W. Walker, Robert C. Kaplan, Qibin Qi

**Affiliations:** a Department of Epidemiology and Population Health, Albert Einstein College of Medicine, Bronx, NY, USA; b Department of Environmental Medicine, Icahn School of Medicine at Mount Sinai, New York, NY, USA; c Division of Public Health Sciences, Fred Hutchinson Cancer Research Center, Seattle, WA, USA

**Keywords:** Added sugar, gut microbiota, inflammation, short-chain fatty acid, amino acid metabolism, cardiometabolic health

## Abstract

Excessive intake of added sugars is a global public health concern, given its established links with cardiometabolic disease and other chronic conditions. Emerging evidence suggests that the gut microbiota might mediate the harms of high sugar intake. In this review, we summarize evidence from animal and human studies regarding the impact of added sugar intake on gut microbiota diversity and composition, and discuss potential mechanisms linking sugar-induced microbial changes to health outcomes. Added sugars, including glucose, fructose, and sucrose, can alter gut microbial diversity, enrich sugar-utilizing taxa, and deplete short-chain fatty acid-producing bacteria. These microbial changes may impair gut barrier integrity, increase luminal oxygen and alternative electron acceptors under inflammatory conditions, reduce short-chain fatty acid production, alter bile acid and amino acid metabolism, and promote translocation of endotoxin across the gut barrier into the bloodstream. Collectively, these pathways may link added sugar intake to irritable bowel syndrome, obesity, liver steatosis, diabetes, and cardiovascular diseases. However, inconsistent results on alterations in the gut microbiota related to added sugar intake were observed across studies, which may be due to differences in sugar dose and form (liquid vs. solid), as well as population variation in background diet, host genetics, and gut microbial ecology. Future research should focus on mechanistic investigations, characterization of inter-individual variability in response to added sugar intake, and clinical studies to assess whether dietary or therapeutic interventions can reverse sugar-induced gut microbial changes and improve host health outcomes.

## Introduction

In industrialised societies, monosaccharides and disaccharides are often added to foods and beverages during processing or preparation.^1^ Distinct from those found naturally in foods and beverages, these added sugars confer disease-promoting effects and have distinctive nutritive values.^2^ Naturally occurring sugars are typically consumed within whole foods (e.g., fruits, vegetables, and dairy), where fibre, water, micronutrients, and phytochemicals slow sugar absorption and modulate postprandial responses.^2^ In contrast, added sugars lack these protective components and are usually consumed in refined and concentrated forms, leading to faster absorption, larger glycemic and insulinemic excursions, and greater amounts of sugars reaching the distal intestine.^2^^,^^3^ These differences create a distinct ecological and metabolic challenge to the gut microbiota. Excess intake of added sugars has been consistently linked to a range of adverse health outcomes, including but not limited to obesity, metabolic syndrome, type 2 diabetes, cardiovascular disease, liver disease, gout, certain cancers, dental caries, depression, and mortality.^1^

In response to these health risks, policies, regulations, and product reformulations have been implemented to alter the use of added sugars in food processing and reduce their consumption.^4^ Strategies employed in various regions include taxation, enhanced nutrition labelling, vending machine restrictions, and limits on marketing to children.^4^ Also, clear dietary recommendations of limiting added sugar intake to less than 10% of total daily energy have been issued by World Health Organisation^5^ and dietary guidelines from the US,^6^ Canada,^7^ China,^8^ Nordic countries,^9^ and elsewhere. These efforts have contributed to declining added sugar consumption in several high-income countries over recent decades.^10-12^ However, the mean intake level is still above 10% of total daily energy in the US (2017−2018)^10^^,^^11^ and around 10% in the UK (April 2016-January 2019).^12^ On the other hand, there is an increasing trend in added sugar intake in other countries (e.g., China,)^13^ potentially due to the increased availability, affordability, and acceptability of sugar-sweetened beverages (SSBs).^14^ Notably, substantial demographic variations in added sugar intake have been observed. In the 2017−2018 US National Health and Nutrition Examination Survey (NHANES), adolescents (9−18 years) consumed a mean of 14.3% of total daily energy from added sugars, higher than children aged 2−8 years (12.6%) and adults (12.4%);^10^^,^^11^ among adults, non-Hispanic Black individuals had higher added sugar intake than non-Hispanic White and Hispanic individuals, whereas Asian American individuals had the lowest intake levels;^11^ additionally, adults with lower family income had higher added sugar intake.^11^ These demographic-specific dietary patterns should be considered when evaluating the health impacts of added sugar consumption.

Beyond efforts to reduce added sugar intake, it is equally important to understand the biological mechanisms linking added sugar intake to health risk to devise intervention targets. High sugar intake, especially fructose intake, has been linked to alterations in fat metabolism,^15^ inflammation,^16^ hyperinsulinemia,^17^ insulin resistance,^15^^,^^17^ and appetite dysregulation.^18^ Moreover, the gut microbiota is increasingly recognised as a key mediator of these effects, given its critical role in maintaining metabolic, immune, and gastrointestinal homoeostasis.^19^ This review integrates evidence from both human and animal studies to elucidate how added sugar intake influences the gut microbiota and, in turn, affects host health.

### Added sugar intake and gut microbial diversity

Rat, mouse, and human studies have examined the influences of added sugar intake on gut microbial diversity. A common metric to characterise the gut microbiome is alpha-diversity, which refers to within-sample diversity considering factors including richness (the number of different organisms) and evenness (how evenly distributed these organisms are in terms of abundance).^20^ Previous animal and human studies compared alpha-diversity between study subjects with different added sugar intake levels. Some reported that the gut microbial richness and evenness decreased after consuming fructose,^21-23^ glucose,^22^^,^^24^ and sucrose^21^^,^^25^^,^^26^ (Tables 1 and 2), while others found no significant changes,^26-45^ and some even observed increased gut microbial diversity after sucrose intake.^21^^,^^46^^,^^47^

Though not fully understood, the inconsistent findings likely arise from multiple factors, including the varied study designs (Tables 1 and 2). First, added sugars from different food sources can have different impacts on the gut microbiome.^44^^,^^48^ C57BL/6J mice consuming a solid diet with 60% of energy from fructose had a higher alpha-diversity than those receiving the same proportion of energy from fructose syrup solution.^48^ Also, in a study among US Hispanic/Latino adults, fructose and glucose intake from beverages, but not from solid foods, was associated with abundances of specific gut bacteria.^44^ Second, gut microbial responses vary by added sugar doses.^49^ For instance, a rat study reported that high and medium fructose doses (5.3 and 10.5 g fructose/kg body weight/day) produced different microbial shifts after 20 weeks compared with a low dose (2.6 g fructose/kg body weight/day). Third, background diets might influence results,^43^^,^^46^ as dietary factors have been found to modify the associations between added sugar intake and gut microbiota. A 10-week sucrose intervention (30 grams of sucrose per 100 mL water) increased the Shannon index in male C57BL/6J mice fed a normal-fat diet but decreased it when fed a high-fat diet.^46^ Similarly, distinct gut microbial features were linked to fructose intake when mice were maintained on control versus western-style diets.^43^ Fourth, host genetics may modulate gut microbial responses.^31^^,^^50^ For example, an 8% fructose solution altered overall caecal microbiota composition in DBA/2J (a fructose-sensitive strain) but not in C57BL/6J (a fructose-resistant strain) mice.^51^ Also, a mouse study demonstrated that *Fut2* gene modifies microbial responses to glucose intake.^50^ *Fut2* gene encodes an α1−2 fucosyltransferase that adds terminal fucose residues to intestinal mucins (i.e., mucosal fucosylation). In humanised gnotobiotic C57BL/6J mice maintained on a standard polysaccharide-rich, glucose-deficient diet, Fut2^−^ mice that lack mucosal fucosylation had lower gut microbial alpha-diversity and enriched *Parabacteroides*, *Bacteroides*, and *Parasutterella* compared with Fut2^+^ mice, while these genotype-dependent differences disappeared under a glucose-rich diet.^50^ This *Fut2*-glucose interaction likely reflects the ability of some gut bacteria to utilise both fucose and glucose as carbohydrate sources^52^—when dietary glucose is abundant, these bacteria use glucose and reduce their reliance on host mucosal fucose, reducing abundance differences in these gut bacteria between host genotypes; while under glucose-restricted conditions, mucosal fucose becomes a major carbohydrate source, and the growth of these bacteria is inhibited in mice lacking mucosal fucosylation. These findings highlight that the host glycan landscape and dietary sugars jointly shape gut microbial composition, suggesting that genetically determined variation in mucosal fucosylation may partly explain heterogeneous microbial responses to high-sugar diets.

**Table 1.** Study design and observed changes in gut microbial diversity and SCFAs in rat and mouse studies.^1^

Author (year)	Animal	Intervention approach	Control group and maintenance diet	Microbiome profiling	Changes in diversity or SCFAs
Ahn^51^	8-wk male C57BL/6J (B6), DBA/2J (DBA), and FVB/NJ (FVB) mice; *n* = 12 for each strain	ad libitum access to water and feed; 8% (w/v; weight of solute in grams per volume of solution in mL) fructose dissolved in regular water for 1, 2, 4, and 12 wks.	Lab Rodent Diet 5001 and regular water; maintenance diet: protein 28%E, fat 12%E, carbohydrate 60%E	16S rRNA (caecal and faecal samples)	Fructose from liquid affected faecal microbial beta-diversity in B3 and DBA (with enriched *Rikenellaceae* and *Pseudomonadaceae*) but not FVB, and affected caecal microbial beta-diversity in DBA (with enriched *Erysipelotrichaceae*) but not B3 or FVB.
Astbury^53^	8-wk Wistar rats; *n* = 30 for Gen0 and 20 for Gen1	ad libitum access to water and feed; 10% fructose water from 2 wks before mating until delivery (6 wks; both Gen0 and Gen1).	LabDiet 5001 + distilled water; maintenance diet: protein 28%E, fat 12%E, carbohydrate 60%E	16S rRNA (faecal samples)	Fructose from liquid affected beta-diversity, with depleted *Bacteroidetes*.
Bergentall^29^	11-to−17-wk male C57BL/6 mice; *n* = 17	ad libitum access to water and feed; sucrose-rich zero-fat diet (0%E fat, 24.2%E protein, 75.8%E sucrose) for 3 wks	Autoclaved chow diet (LabDiet); maintenance diet: protein 28.9%E, fat 13.6%E, carbohydrate 57.5%E	16S rRNA (caecum samples)	Sucrose from solid food did not change diversity but affected beta-diversity.
Brütting^54^	4-wk male C57BL/6J mice; *n* = 48	ad libitum access to water and feed; two-factor design (4 groups): F+ received a diet that contained 40% fructose; P+ received a diet that contained 1% propionate; intervention lasted for 7 wks.	Basal diet; maintenance diet: protein 19.5%E, fat 21.9%E, carbohydrate 58.6%E from maize starch, sucrose, and maltodextrin	16S rRNA (ileum and colon contents; *n* = 6 for each group)	Fructose from solid food did not change colonic acetic acid, propionic acid, or butyric acid.
Chen^21^	4-wk male C57BL/6J mice; *n* = 30	ad libitum access to water and feed; 30% (w/v) fructose or sucrose in drinking water for 16 wks.	Standard chow diet + tap water; maintenance diet: protein 20.3%E, fat 15.8%E, carbohydrate 63.9%E	Shotgun (faecal samples)	Fructose and sucrose from liquid increased richness while decreasing diversity. Fructose and sucrose affected beta-diversity. Fructose enriched *Lactobacillus*, *Bacteroidales* gen., *Muribaculaceae gen.*, *Morganelia,* and *Muribaculum*. Sucrose enriched *Enterobacter*, *Enterobacteriaceae* gen., *Klebsiella*, *Enterobacterales* gen., and *Escherichia*.
Chen^55^	4-wk male C57BL/6J mice; *n* = 20	ad libitum access to water and feed; 30% (w/v) FCS (D-(+)-glucose:D-(-)-fructose is 45:55) in drinking water for 16 wks.	Standard chow diet + tap water; no information on maintenance diet	Shotgun (faecal samples)	HFCS from liquid increased diversity and affected beta-diversity, with decreased *Firmicutes* to *Bacteroidetes* ratio and enriched *Deferribacteres* and *Verrucomicrobia*.
Crescenzo^56^	14-wk male SD rats; *n* = 24	ad libitum access to water and feed; fructose diet (same macronutrient energy distribution as the control diet, with 30%E from fructose, 22.8%E from starch, and 7.6%E from sugars) for 8 wks; quantities of feed were identical between two groups.	Control diet; maintenance diet: protein 29.0%E, fat 10.6%E, carbohydrate 60.4%E from starch and sugars without fructose	16S rRNA (caecal contents)	Not reported
de Oliveira Neves^30^	4-wk male Wistar rats; *n* = 32	ad libitum access to water and feed; high-sugar diet (260.01 g of added sugar per kg) for 15 wks	Standard rat chow (Nuvilab CR1®, Colombo, Brazil); maintenance diet: protein 28.09%E, fat 14.75%E, carbohydrate 57.16%E	16S rRNA (faecal samples)	Added sugar from solid food did not change diversity.
Do^22^	8-wk male C57BL/6J mice; *n* = 36	ad libitum access to water and feed; 65% of calories in carbohydrate (85% from glucose or fructose and 15% from sucrose) for 12 wks.	Normal diet; maintenance diet: protein 24.0%E, fat 18.0%E, carbohydrate 58.0%E	16S rRNA (faecal samples)	Fructose and glucose from solid food decreased richness and diversity.
Fu^31^	7-wk male C57BL/6N, C3H/HeJ, DBA/2, ICR, and ddY mice; *n* = 10 for each strain	ad libitum access to water and feed; high-sucrose diet (isocaloric to the control starch diet, which substituted all starch with sucrose [67.6%E]) for 4 wks.	Control starch diet; maintenance diet: protein 20.7%E, fat 11.7%E, carbohydrate 67.6%E	16S rRNA (caecum content)	Sucrose from solid food did not change richness or diversity, but affected beta-diversity. Sucrose from solid food did not change colonic formic acid, acetic acid, propionic acid, butyric acid, or succinic acid.
Gao^32^	4-wk female SD rats; *n* = 30	ad libitum access to water and feed; fructose 10% and 20% w/vol, glucose 10% w/vol for 10 wks.	AIN-93G; maintenance diet: protein 20%E, fat 16%E, carbohydrate 64%E	16S rRNA (colon content)	Fructose from liquid did not change richness or diversity but affected beta-diversity, with enriched *Allobaculum* and *Lactobacillus*, as well as depleted *Lachnospiraceae* and *Ruminococcaceae*. Glucose from liquid did not change richness, diversity, or beta-diversity.
He^33^	5-wk male C57BL/6J mice; *n* = 24	ad libitum access to water and feed; drinking water containing 30% sucrose for 24 wks.	Chow diet; maintenance diet: protein 22.8%E, fat 13.8%E, carbohydrate 63.4%E	16S rRNA (caecal content)	Sucrose from liquid did not change richness, diversity, or beta-diversity.
Hrncir^34^	3-wk germ-free C57BL/6J mice colonised by healthy human faecal samples; *n* = 40	ad libitum access to water and feed; fructose (10% fructose [w/v] in water) for 11 wks.	Water (control) group; maintenance diet: protein 27%E, fat 12%E, carbohydrate 61%E	16S rRNA (no specimen information)	Fructose from liquid did not change richness or diversity but affected beta-diversity, with enriched *Pastescibacteria* and depleted *Proteobacteria*.
Hsu^35^	Pregnant SD rats (*n* = 12) and male offspring (fed a normal diet; *n* = 31)	ad libitum access to water and feed; high-fructose diet (60% of diet by weight) for 6 wks in G0 (during pregnancy and lactation).	Nomal diet; maintenance diet: protein 27.9%E, fat 14.1%E, carbohydrate 58.0%E	16S rRNA (faecal sample)	Fructose from solid food did not change diversity but affected beta-diversity, with depleted *Verrucomicrobia*. Fructose from solid food increased blood acetic acid but did not change blood propionic acid or butyric acid.
Jegatheesan^57^	Male SD rats; *n* = 58	ad libitum access to access to water and standard rodent chow; mean fructose intake in the fructose group is 5.3 g/100 g BW/d for 4 wks.	Standard rodent chow; maintenance diet: protein 19.3%E, fat 8.2%E, carbohydrate 72.5%E	qPCR (caecal contents)	Not reported
Kashyap^50^	12-wk germ-free C57BL/6J mice humanised by oral gavage of a human (American diet) faecal sample; *n* = 28	Irradiated custom diet containing 68% glucose w/v (66.8%E), 18% protein w/v (17.7%E), and 7% fat w/v (15.5%E) for 4 wks.	Autoclaved polysaccharide rich standard diet (Purina LabDiet 5K67); maintenance diet: protein 22.1%E, fat 16.3%E, carbohydrate 61.6%E from starch and sucrose	16S rRNA (faecal sample)	Glucose from solid food affected beta-diversity, with enriched *Parabacteroides, Bacteroides,* and *Parasutterella* in humanised Fut2 + mice.
Kim^24^	4-wk male C57BL/6J mice; *n* = 50	ad libitum access to water and feed; dissolved dextrose in distilled water (20% final concentration) for 2 or 5 wks; GL261 tumour cells were inoculated at wk 5.	Control diet; maintenance diet: protein 24.2%E, fat 17.7%E, and carbohydrate 58.1%E	16S rRNA (faecal samples)	Glucose from liquid decreased richness.
Mastrocola^48^	4-wk male C57BL/6J mice; *n* = 30	Fructose liquid (L-Fr): 60% fructose (w/v) syrup solution; Fructose solid formulation (S-Fr): 70%E from carbohydrates (60%E from fructose), 10%E from fat, and 20%E from proteins. Both were administered for 12 wks.	Standard diet + tap water; maintenance diet: protein 17.6%E, fat 5.1%E, carbohydrate 67.1%	16S rRNA and shotgun (ileum content)	Not reported
Mhd Omar^37^	7-wk male SD rats; *n* = 90	ad libitum access to water and feed; high-carbohydrate diets consisted of 50% fructose or 50% galactose (isocaloric to control starch diet; mean daily intake: 15.2 g for fructose and 14.0 g for galactose) for 6 and 12 wks.	Control diet; maintenance diet: 50% starch as the carbohydrate source and 6% safflower oil as a source of *n*-6 PUFAs	16S rRNA (intestinal sample contents)	Fructose from solid food did not change richness or diversity.
Min^58^	12-wk male SD rats; *n* = 30	The oral high glucose (OHG) group was fed with 50% high glucose at a dose of 2.5 g/kg/day. The high glucose infusion (IHG) group was treated with 50% high glucose via tail vein injection at a dose of 2 g/kg/day. Both interventions lasted for 2 wks.	An equivalent amount of saline orally; maintenance diet: standard chow without information on macronutrient composition	16S rRNA (faecal samples)	Not reported
Montrose^59^	C57BL/6J male mouse colitis model by DSS; *n* = 17	ad libitum access to water and feed; 62.9 g of fructose per 100-g food (almost in an isocaloric way; high fructose diet substitutes control diet's corn starch and maltodextrin, but not sucrose) for 2 wks; foods providing 15%E from fructose were used as an exploratory experiment; DSS was administered on day 8.	AIN-93G purified diet; maintenance diet: protein 20%E, fat 16%E, carbohydrate 64%E mostly from corn starch, maltodextrin, and sucrose	16S rRNA (faecal samples)	Not reported
Oh^60^	7-wk male C57BL/6J mice; *n* = 24	ad libitum access to water and feed; drinking water containing either glucose or fructose (14 mg/mL) for 1 wk.	Standard chow (LabDiet 5008); maintenance diet: protein 28.9%E, fat 13.6%E, carbohydrate 57.5%E	Not reported	Not reported
Pessoa^38^	Adult male C57BL/6 mice; *n* = 46	ad libitum access to water and feed; drinking water supplemented with a 30% (w/v) mixture of 55/45% fructose/glucose for 9 and 18 wks.	Standard chow diet; maintenance diet: protein 19.2%E, fat 9.1%E, carbohydrate 71.7%E (5.7%E from sucrose)	16S rRNA (faecal samples)	HFCS from liquid did not change richness or diversity but affected beta-diversity, with depleted *Akkermansia* (wks 9 and 18), *Paludicola* (wk 18), *Eisenbergiella* (wk 18), and *Ruminococcaceae Incertae sedis* (wk 18), as well as increased *Anaerovorax* (wk 9), ASF356 (wk 9), and *Clostridium Sensu Stricto* 1 (wks 9 and 18).
Peterson^39^	4-to−6-wk male C57BL/6 mice; *n* = 16	ad libitum access to water and feed; 649.19 g fructose/kg diet for 6 wks.	Standard mouse chow; no information on maintenance diet	Shotgun (faecal samples)	Fructose from solid food did not change richness but affected beta-diversity, with enriched *Bacteroides* and *Lactococcus*, as well as depleted *Muribaculum* and *Duncaniella*.
Ramos-Romero^61^	8-to−9-wk male WKY rats; *n* = 27	ad libitum access to water and feed; 35% (w/v) sucrose in mineral water for 24 wks.	Standard diet + mineral water; maintenance diet: protein 20%E, fat 13%E, carbohydrate 67%E	qPCR (faecal contents)	Sucrose from liquid did not change faecal acetic acid, propionic acid, butyric acid, or total SCFA.
Sen^25^	6-wk male SD rats; *n* = 12	ad libitum access to water and feed; Low-fat/high-sucrose diet (LF/HSD): protein 20.0%E, fat 10.0%E, carbohydrate 70.0%E (with 17.0%E from sucrose); high-fat/high-sucrose diet (HF/HSD): protein 20.0%E, fat 45.0%E, carbohydrate 35.0%E (with 17.0%E from sucrose); the intervention lasted for 4 wks.	Lab Diet PicoLab Rodent Diet 20 (5053; LF/LSD); maintenance diet: protein 24.5%E, fat 13.1%E, carbohydrate 62.4%E (with 3.2%E from sucrose)	16S rRNA (faecal sample)	Sucrose from solid food decreased diversity and affected beta-diversity, with enriched *Aerococcaceae*, *Desulfovibrionaceae*, *Alcaligenaceae*, *Lachnospiraceae,* and *Ruminococcaceae*, as well as depleted *Prevotellaceae*, *Lactobacillaceae*, *Eubacteriaceae*, *Coriobacteriaceae*, *Rikenellaceae*, *Cerasicoccaceae*, and an unnamed family from *Bacteroidales*.
Shon^46^	5-wk male C57BL/6J mice; *n* = 60	ad libitum access to water and feed; sucrose 30% w/v for 10 wks.	Normal-fat diet (protein 20%E, fat 10%E, carbohydrate 70%E with 7%E from sucrose) or high-fat diet (when compared with High-fat diet + sucrose; protein 20%E, fat 60%E, carbohydrate 20%E with 7%E from sucrose)	16S rRNA (faecal samples)	Sucrose from liquid increased diversity in normal-fat diet group while decreasing diversity in high-fat diet group; it only decreased evenness in high-fat diet group. Sucrose from liquid affected beta-diversity in both fat diet groups. In high-fat diet group, sucrose enriched *Prevotellaceae*, *Bacteroidaceae, Paraprevotellaceae*, and *Enterobacteriaceae,* while depleting *Rikenellaceae*.
Sun^26^	6-wk male Wistar rats; *n* = 40	ad libitum access to water and feed; 100-g foods containing 65.3-g sucrose for 4 wks.	Control starch diet; maintenance diet: protein 20.7%E, fat 11.7%E, carbohydrate 67.6%E	16S rRNA (caecal contents)	Sucrose from solid food decreased richness but did not change evenness. Sucrose from solid food affected beta-diversity, with enriched *Verrucomicriobia* and *Bacteroidetes*, and depleted *Firmicutes*. Sucrose from solid food decreased formic acid and butyric acid but did not change acetic acid, propionic acid, or succinic acid in colonic content.
Suriano^42^	8-wk male C57BL/6J mice; *n* = 55	ad libitum access to water and feed; high-sugar diet (isocaloric to the control diet with 17.2%E from sucrose) for 8 wks.	Low-sugar, low-fat control diet for high-sugar; maintenance diet: protein 20.2%E, fat 10.1%E, carbohydrate 69.7%E (6.9%E from sucrose)	16S rRNA (faecal samples)	Sucrose from solid food did not change richness or diversity, but affected beta-diversity, with enriched *Faecalibaculum* and *Turicibacter*.
Szabó^62^	10-wk male Wistar SPF rats (200−275 g)	ad libitum access to water and feed; per 100-g diet containing 65 g of glucose or fructose for 30 d.	AIN-93G formula (65-g starch per 100-g diet); maintenance diet: protein 17.7%E, fat 14.3%E, carbohydrate 68.0%E	Shotgun (caecal contents)	Not reported
Tain^63^	Male SD rats; *n* = 40	ad libitum access to water and feed; high-fructose diet (fructose 60% of the food weight, providing 66.7%E; protein 23.3%E, fat 12.5%E); the intervention lasted for 9 wks (from 3 wks to 12 wks of age), and the dams were fed the same high-fructose diet for 6 wks during pregnancy and lactation.	Nomal diet; maintenance diet: protein 27.9%E, fat 14.1%E, carbohydrate 58.0%E	16S rRNA (faecal sample)	Not reported
Volynets^43^	8-wk female C57BL/6J mice; *n* = 48	ad libitum access to water and feed; water containing 30% D(-)-fructose for 12 wks.	High-sugar control diet (CD; protein 19%E, fat 13%E, carbohydrate 68%E [43%E from starch, 25%E from sucrose]) or western-style diet (WSD; protein 15%E, fat 43%E, carbohydrate 42%E [13%E from starch, 29%E from sucrose]) + plain tap water	16S rRNA (faecal sample)	Fructose from liquid did not change richness, diversity, or beta-diversity.
Wang^49^	6-wk male SD rats; *n* = 40	0.26 (L), 0.53 (M), and 1.05 g (H) of fructose for 20 wks.	Saline solution; maintenance diet: protein 23.1%E, fat 11.8%E, carbohydrate 65.1%E	16S rRNA (colonic contents)	Fructose from solid food did not change colonic acetic acid, propionic acid, butyric acid, or total SCFA.
Zhang^47^	7-wk C57BL/6 mice; *n* = 50	ad libitum access to water and feed; 7.5 mg/mL sucrose (L), 15 mg/mL (M, corresponding to free sugar intake of 25g/d), 30 mg/mL (H) for 3 wks.	Control chow or Dextran sodium sulphate modelling (colitis model); no information on maintenance diet.	16S rRNA (colonic contents)	Sucrose from liquid increased evenness but decreased richness. Sucrose from liquid affected beta-diversity - L enriched *Acidobacteria, Bacteroidetes,* and *Actinobacteria*, while depleting *Firmicutes* and *Verrucomicrobia*; M and H enriched *Firmicutes, Bacteroidetes, Cyanobacteria*, and *Patescibacteria.* Fructose from liquid increased colonic acetic acid, propionic acid, butyric acid, and total SCFA.
Zhou H^45^^,^^64^	7-wk male ApoE-/- mice; *n* = 44	ad libitum access to water and feed; 227.7 g of sugar per 4000-kcal food (per kg food contains 4119 kcal) for 12 wks; diets from three groups were isocaloric.	Refined and whole grain-enriched diet (whole grain-enriched diet has twice dietary fibre contents than that of the other two diets); maintenance diet: protein 24.7%E, fat 19.8%E, carbohydrate 54.5%E	16S rRNA (faecal samples)	Added sugar from solid food did not change richness, evenness, or diversity, but affected beta-diversity, with increased ratio of *Firmicutes* to *Bacteroidetes*, *Desulfobacterota,* and *Campylobacterota*, as well as depleted *Verrucomicrobia* and *Cyanobacteria*.
Zhou X^23^	7-wk male C57BL/6J mice; *n* = 30	ad libitum access to water and feed; 60 g of fructose (only carbohydrate source) per 100 g of food for 4 wks for gut microbiome and 12 wks for other measurements.	Control diet; maintenance diet: protein 19.4%E, fat 8.1%E, carbohydrate 72.5%E	16S rRNA (faecal samples)	Fructose from solid food decreased richness and diversity and affected beta-diversity, with enriched *Firmicutes* and depleted *Bacteroidetes*.

^1^ Alpha-diversity evaluates richness, evenness, and diversity—richness evaluates the number of different organisms; evenness evaluates how evenly distributed these organisms are in terms of abundance; diversity considers both richness and evenness.

**Table 2.** Study design and observed changes in gut microbial diversity and SCFAs in human studies.^1^

Author (year)	Study design	Country	Population	Age	Sex	Race/ethnicity	Sugar intake	Faecal microbiome profiling	Changes in diversity or SCFAs
Alemán^27^	Double-blind, randomised, cross-over	US	Men and postmenopausal women with BMI of 30−39 kg/m^2^. Exclusion criteria: CVD, diabetes, low liver function, high serum uric acid, some digestive diseases, some infectious diseases, use of probiotics or antibiotics, tobacco smoker, heavy alcohol drinker (*n* = 10).	45−70	Female 40%, male 60%	Black 70%, White 20%, Hispanic 10%	75 g of sugar (fructose or glucose) which replaced 75 g of complex carbohydrates from the usual diet (carbohydrate 53%E, protein 14%E, fat 33%E);14 days for fructose (or glucose), 3 days of wash-out, and 14 days for glucose (or fructose; unknown study time).	16S rRNA	• Fructose and glucose from solid food did not change richness, diversity, or beta-diversity.
Beisner^28^	Open-label, single-arm intervention study	Germany	Healthy female volunteers. Exclusion criteria: Smoking, pregnancy/breastfeeding, chronic gastrointestinal diseases, chronic anaemia, chronic hepatic or renal disease, or diabetes (*n* = 12)	20−40 (mean 28)	Female 100%	White mostly	Wk1: low-fructose diet (fructose < 2%E/d or 10 g/d); wk2: high-fructose diet rich in fruits and vegetables (fructose ~20%E/d or 100 g/d); wk3: low-fructose diet (fructose < 2%E/d or 10 g/d); wk4: high-fructose diet supplemented with high-fructose syrup (HFS, fructose ~20%E/d or 100 g/d). The diet composition is protein 15%E, fat 30%E, and carbohydrate 55%E.	16S rRNA	• HFS did not change diversity.
Jones^65^	ad hoc analysis of two trials	US	Overweight/obese adolescents from two trials (Meta-AIR study and a 16-week parallel, double-blind and placebo-controlled trial examining the efficacy of probiotic supplementation in changing gut microbiota); *n* = 52.	12−19 (mean 17)	Female 44%, male 56%	Hispanic 83%	One or two 24-hour diet recalls (2014−2016). Mean daily intake: added sugar 57.2 g, fructose 24.8 g, glucose 22.9 g	16S rRNA	Not reported
Kwok^36^	Double-blind, randomised, parallel-design	Canada	Non-pregnant, non-lactating adults with a normal BMI (18.5−24.9 kg/m^2^) with intake of ≤600 mL high-intensity sweetened beverage per week for a month and a normal bowel frequency (*n* = 59). Exclusion criteria: Use of probiotics or antibiotics, non-smoking, high alcohol consumption, history of major trauma or major medical/surgical event, diabetes, bariatric surgery, phenylketonuria, some digestive diseases, renal failure, some liver diseases, or weight change of >5 kg in the preceding 3 months.	18−50 (mean 31)	Female 61%, male 39%	Asian 42%, White 37%, Hispanic 12%, multi-racial 7%, Black 2%	One 16-oz beverage containing 30 g of sucrose or 75.6 mg steviol (which is not of interest in this review) daily for 4 weeks (2022)	Shotgun metagenomic sequencing (2022)	Sucrose from liquid did not change richness, diversity, or beta-diversity. Sucrose from liquid did not change faecal acetic acid, propionic acid, butyric acid, or succinic acid.
Latorre-Pérez^66^	Cross-sectional survey	Spain	Healthy Spanish adults (*n* = 530). Exclusion criteria: BMI ≥ 35 kg/m^2^; use of probiotics (large dose, commercial), antibiotics, corticosteroids, cytokines, methotrexate, or immunosuppressive cytotoxic agents; acute illness; chronic lung, cardiovascular, gastrointestinal, liver, or kidney function abnormalities; cancer; unstable dietary history; chronic alcohol use; HIV, HBV, HCV, or immunosuppression/immunodeficiency condition/status; pregnant or nursing women.	18−70	Female 50%, male 50%	Hispanic 100%	Dietary habits questionnaire (2019−2020) to obtain SSB intake	16S rRNA	Not reported
Mokhtari^67^	Cohort (Southern California Mother’s Milk Study)	US	6-month infants (*n* = 105). Exclusion criteria: Inflammatory/infectious disease, fatal abnormalities, pre-term/low birth weight, mothers < 18 years of age at delivery, and pregnancy complications	0.5	Female 54%, male 46%	Hispanic 100%	Three 24-hour dietary recalls (2016–2019). Mean daily intake: added sugar 14.3 g, free sugar 5.0 g, total sugar 61.8 g.	16S rRNA (2016−2019)	Not reported
Ramne^40^	Cohort (Malmö Offspring Study)	Sweden	Adult children and grandchildren of participants in the Malmö Diet and Cancer-Cardiovascular Cohort, who were free from diabetes (*n* = 1086).	mean 42	Female 57%, male 43%	White mostly	A combination of 4-day food records and FFQ (2017)	16S rRNA	• Added sugar from liquid did not change diversity, but affected beta-diversity, with depleted *Lachnobacterium* and increased *Firmicutes* to *Bacteroidetes* ratio.
Senaprom^41^	Open-label, randomised, parallel-design	Thailand	Adults aged 18−45 y, with normal BMI (18.5−22.9 kg/m^2^), waist circumference ( ≤ 90/80 cm for men/women), blood pressure (SBP/DBP ≤ 120/80 mmHg), and pulse (60−100 times/min); *n* = 30. Exclusion criteria: Use of antibiotics, prebiotics, or probiotics, smoking during the last 3 months, alcohol consumption during the last week, and gastrointestinal disease/diarrhoea during the last week.	18−45 (mean 29)	Female 83%, male 17%	Asian mostly	After an 8-to−12-h fasting, participants consumed phetchaburi’s Custard Cake (low-GI dessert group; 192 g), Saraburi’s Curry Puff (medium-GI dessert group; 98 g), or Lampang’s Crispy Rice Cracker (high-GI dessert group; 68 g), with 150 mL of still water and within 15 min; all desserts were portioned to provide 50 g of available carbohydrate content, with sucrose as the major sugar source. (2023)	16S rRNA	• Sucrose from solid food did not change richness, diversity, or beta-diversity.
Zhang^44^	Cohort (Hispanic Community Health Study/Study of Latinos)	US	US Hispanic/Latino adults (*n* = 2970)	18−74 (mean 45.8)	Female 60%, male 40%	Hispanic 100%	Two 24-hour dietary recalls (2008−2011). Mean daily intake: SSB 1 serving (8-oz), fructose 23.0 g, glucose 22.2 g, sucrose 42.6 g.	Shotgun metagenomic sequencing	• Added sugar from liquid did not change richness, diversity, or beta-diversity.

^1^ Alpha-diversity evaluates richness, evenness, and diversity—richness evaluates the number of different organisms; evenness evaluates how evenly distributed these organisms are in terms of abundance; diversity considers both richness and evenness.

Beta-diversity refers to between-sample diversity, i.e., differences in overall microbiota composition between individuals with different added sugar intake levels.^20^ With a few exceptions,^27^^,^^32^^,^^33^^,^^36^^,^^43^^,^^44^^,^^51^ most studies reported that fructose,^21^^,^^23^^,^^32^^,^^34^^,^^35^^,^^39^^,^^51^^,^^53^ glucose,^50^ sucrose,^21^^,^^25^^,^^26^^,^^29^^,^^31^^,^^41^^,^^42^^,^^46^^,^^47^ high-fructose corn syrup (HFCS),^38^^,^^55^ and added sugar intake,^40^^,^^45^ significantly altered gut microbial community structure.

### Gut microbiota related to added sugar intake

**Figure 1.**
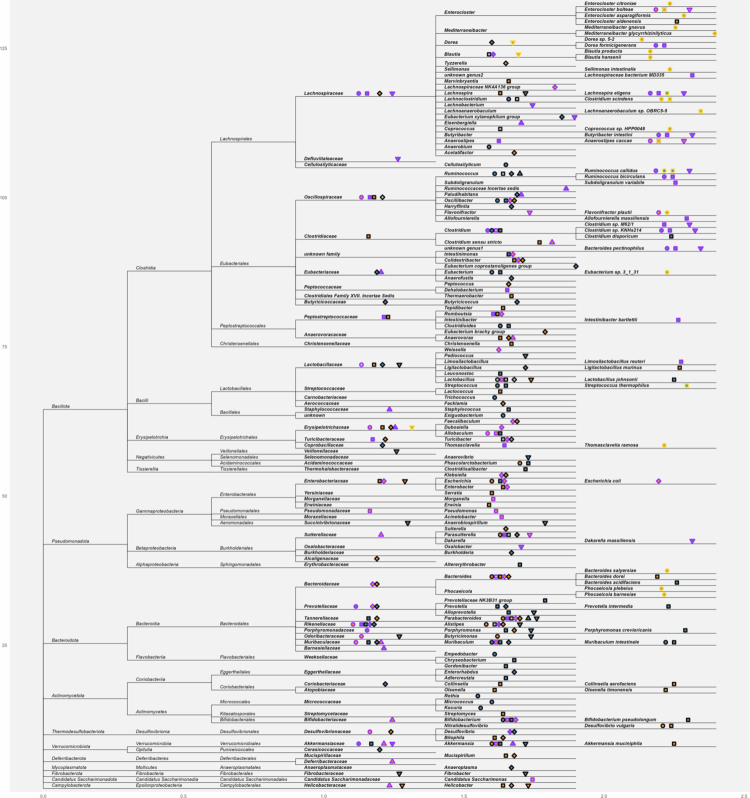
Gut microbiota related to added sugar intake: animal and human evidence. Markers following taxa names indicate intervention methods and association directions. Shapes indicate different sugar types: circles for glucose, squares for fructose, diamonds for sucrose, upright triangles for high-fructose corn syrup, and inverted triangles for added sugars. Border colours indicate food sources: purple for liquid, black for solid food, and yellow for both liquid and solid food. Fill colours indicate the directions of associations: orange for positive, blue for inverse, and grey for mixed associations.

Current mammalian evidence consistently observed several gut bacteria to be enriched by added sugar intake (Figure 1 shows sugar-related taxonomy at or below the family level), including *Blautia*,^23^^,^^47^^,^^49^^,^^67^^,^^68^ *Anaerovorax*,^23^^,^^29^^,^^38^ *Faecalibaculum*,^33^^,^^42^^,^^47^ *Turicibacter*,^35^^,^^42^^,^^47^ *Bacteroides*,^23^^,^^29^^,^^32^^,^^39^^,^^44^^,^^46^^,^^47^^,^^50^^,^^62^ *Helicobacteraceae* (particularly *Helicobacter*),^23^^,^^29^^,^^30^^,^^45^^,^^55^ and *Enterobacteriaceae* (including *Enterobacter*, *Escherichia*, and *Klebsiella*).^21,30,35,41,46,61–63^ Conversely, added sugar intake generally depletes gut *Streptococcus*,^54^^,^^62^^,^^65^ *Clostridium*,^32^^,^^44^^,^^51^^,^^56^^,^^59^^,^^62^ *Eubacteriaceae* (including *Anaerofustis* and *Eubacterium*),^25^^,^^29^^,^^55^^,^^62^ *Ruminococcus*,^28^^,^^29^^,^^44^^,^^62^^,^^68^ *Lachnospira*,^30^^,^^44^^,^^65^^,^^66^ *Muribaculum*,^22^^,^^23^^,^^29^^,^^39^^,^^62^ and *Porphyromonadaceae* (particularly *Porphyromonas*).^24^^,^^30^^,^^39^ Although mechanisms are not fully established, several pathways (e.g., direct sugar utilisation, cross-feeding interactions, and inflammation-mediated ecological shifts) likely contribute to these associations.

#### **Direct sugar utilisation**

Carbohydrate utilisation experiments have shown that many bacteria can ferment sugars. For example, most *Blautia* strains can ferment glucose, and some strains can utilise sucrose and fructose;^69^ certain *Faecalibaculum* species can ferment sucrose.^70^ *Enterobacteriaceae* have diverse sugar transport systems, such as ATP-Binding Cassette (ABC) transporters and phosphoenolpyruvate-dependent Phosphotransferase Systems (PTS),^71^^,^^72^ enabling efficient uptake and metabolism of multiple sugars. These features provide a competitive advantage in sugar-rich environments. By contrast, some rat/mouse experiments substituted starch with simple sugars in an (approximately) isocaloric way,^31^^,^^37^^,^^50^^,^^56^^,^^59^ and microbiota specialised in starch utilisation (e.g., *Ruminococcus*)^73^ declined thereafter, which might be attributed to reduced substrate availability.

#### **Cross-feeding interactions**

Cross-feeding is a process of microbial metabolic cooperation, whereby the fermentation products or metabolites released by one taxon are utilised as growth substrates by another taxon, shaping microbial community structure and function in the gut.^74^ In the context of added sugar intake, simple sugars can be fermented by sugar-utilising bacteria, increasing the availability of fermentation products such as formate, lactate, acetate, and pyruvate.^74^^,^^75^ Bacteria including *Blautia*,^76^ *Bacteroides*,^77^^,^^78^ and *Helicobacter*^79^ can use these fermentation products as substrates, promoting their expansion in sugar-rich ecosystems. On the other hand, excessive sugar consumption can drive substantial lactate production in the colon,^80^ lowering luminal pH, which can down-regulate the proteolytic activity of asaccharolytic *Porphyromonas* and inhibit its growth.^81^^,^^82^

#### **Inflammation-mediated ecological shifts**

Excessive sugar intake has been shown to increase colonic expression of inflammatory cytokine genes (IL-6, TNF-*α*, and IL-1β) in the colon,^47^ alongside histological changes including worsened colitis,^59^ increased villus width,^55^ and increased intestinal wall thickness in the jejunum.^55^ Under physiological conditions, the gut lumen remains largely anaerobic through several mechanisms. Epithelial cells consume oxygen through mitochondrial *β*-oxidation of fatty acids, primarily fuelled by short-chain fatty acids (SCFAs) such as butyrate, thereby creating an oxygen sink that limits oxygen diffusion into the lumen.^83^ The mucus layer further restricts oxygen penetration, and facultative anaerobes near the mucosal surface help scavenge residual oxygen.^83^ However, during inflammation, a disruption of the intestinal microvasculature reduces oxygen delivery to epithelial cells, leading to impaired mitochondrial fatty acid oxidation and reduced epithelial oxygen consumption. Consequently, more unconsumed oxygen diffuses toward the intestinal lumen.^83^ In addition, inflammation increases the fluidity of intestinal contents, facilitating faster oxygen diffusion across the lumen.^83^ Together, inflammation induced oxygen leakage from the epithelium into the intestinal lumen, which favours facultative anaerobes (e.g., *Enterobacteriaceae*)^84-86^ and microaerophiles (e.g., *Helicobacter*),^87^ and suppresses obligate anaerobes (e.g., the majority of *Clostridium* spp.,^88^ *Eubacterium*,^89^ *Ruminococcus*,^90^ and *Porphyromonas*).^91^ Additionally, inflammation-associated induction of nitric oxide synthase generates nitrate,^92^ an alternative electron acceptor that *Enterobacteriaceae* can utilise for anaerobic respiration, enabling greater energy production compared to fermentation and thus fostering their expansion.^93-95^

Despite these consistent findings, previous studies in rats, mice, and free-living humans reported inconsistent changes in the gut abundance of *Bifidobacterium*, *Lactobacillus*, *Oscillibacter*, *Dorea*, *Parabacteroides*, *Parasutterella*, and *Akkermansia* after added sugar intake, suggesting that multiple pathways are involved.

For example, although certain *Bacteroides* species (e.g., *B. fragilis* and *B. thetaiotaomicron*) can ferment glucose and fructose,^77^^,^^96^ and cross-feeding through sugar-fermented lactate and acetate can further support their growth,^77^^,^^78^ two mouse studies reported that fructose intake reduced the relative abundances of genus *Bacteroides* at the genus level and specifically decreased *B. acidifaciens*.^34^^,^^59^ In fact, utilisation of complex polysaccharides is a hallmark of *Bacteroides* ecology—up to ~20% of *Bacteroides* genomes encode polysaccharide utilisation loci (PULs) that enable the sensing, uptake, and degradation of dietary and host-derived complex glycans.^97^ Besides, dietary fructose and glucose repress the production of Roc (a protein essential for gut colonisation by *B. thetaiotaomicron*) by silencing its master regulator BT4338,^98^ suggesting that simple sugars may inhibit the growth or colonisation in certain *Bacteroides* species.

Similarly, glucose and fructose intake from solid food was linked to depleted gut *Bifidobacterium*,^27^^,^^39^^,^^57^ possibly due to oxygen leakage and increased alternative electron acceptors during gut inflammation,^47^^,^^55^^,^^59^^,^^92^^,^^99^^,^^100^ which inhibit oxygen-(hyper)sensitive *Bifidobacterium* species.^99^ However, three rat/mouse studies reported that liquid fructose, sucrose, and HFCS intake increased gut *Bifidobacterium* abundance.^47^^,^^51^^,^^55^ This might be because large doses of liquid fructose with rapid gastric emptying can exceed the absorptive capacity of the small intestine and result in a greater proportion of unabsorbed fructose reaching the colon.^101^ Many *Bifidobacterium* species ferment fructose using the fructose−6-phosphate phosphoketolase enzyme,^102^ and increased colonic fructose availability may therefore provide a direct growth substrate to favour *Bifidobacterium* proliferation. Of note, given the challenging sampling techniques, there is a paucity of data on the small intestine microbiota,^37^^,^^48^^,^^54^ particularly among humans, and our understanding of microbial responses in this region remains limited.

*Akkermansia muciniphila* illustrates further complexity. This mucin-degrading bacterium can also use a variety of sugars including glucose,^103^ meaning added sugars may directly provide substrates for its growth. However, added sugars also induce gut inflammation,^47^^,^^55^^,^^59^ which reduces mucin quality and quantity,^104^ potentially inhibiting this bacterium.

Differences between different members within a single taxonomy add further heterogeneity. For example, glucose and sucrose intake may differentially impact *Desulfovibrionaceae* members. Overall, glucose and sucrose interventions enrich *Desulfovibrionaceae*,^24^^,^^25^ as well as *Bilophila*^23^^,^^25^^,^^29^ and *Desulfovibrio vulgaris*^22^^,^^39^ (under genus *Nitratidesulfovibrio*), while *Desulfovibrio* was depleted by sucrose intervention.^29^^,^^33^ Mechanistic studies suggest that this divergence may reflect distinct ecological niches. Mouse experiments reported that a high-fructose diet increased faecal taurine-conjugated bile acids,^59^ and taurocholic acid, a taurine-conjugated bile acid, can promote *Bilophila* expansion.^105^ Meanwhile, added sugars can be fermented into lactate and hydrogen in the colon, and *D. vulgaris* uses them as electron donors for sulphate reduction and promotes its growth.^106^ In contrast, other gut bacteria from *Desulfovibrionaceae* may be competitively excluded or sensitive to acidic environments generated by excess sugar fermentation.

In addition, two non-mammalian studies in green iguanas^107^ and geese^108^ used 16S rRNA gene sequencing to examine gut microbiota composition following added sugar interventions. In juvenile male green iguanas, a 30-day intervention using tortoise pellets soaked in dextrose water did not change gut microbial alpha-diversity but changed beta-diversity, with depleted *Burkholderiaceae* and *Enterobacteriaceae*.^107^ In Tianfu meat geese, 10-gram maize flour (per 100 grams of diet) was replaced with 10-gram glucose, fructose, and sucrose, respectively.^108^ After 18 days of intervention, fructose supplementation decreased the Chao1 index, indicating reduced richness, and all three sugars enriched jejunal *Lactobacillus*, while only sucrose enriched caecal *Bacteroides*.^108^

### How gut microbiota links added sugar intake to host health

Human gut microbiota participates in energy and nutrient extraction, biosynthesis of bioactive molecules, and immune modulation, thereby influencing human health.^19^ Understanding how added sugar intake influences host health via gut microbiota is essential, as accumulating rat, mouse, and human studies have suggested multiple mechanistic pathways.^21,23,24,26,28,29,31,33,44–47,51,56,59,60^

#### **Intestinal permeability and inflammation**

Rat and mouse studies have observed that added sugar intake decreased the expression of tight junction proteins (e.g., ZO-1 and occludin) in the colon^22^^,^^25^^,^^49^ and increased intestinal permeability,^43^ showing multiple gut inflammatory signs (e.g., worsened colitis,^59^ increased villus width,^55^ and increased intestinal wall thickness in the jejunum).^55^ Increased intestinal permeability and inflammation are features of irritable bowel syndrome, especially diarrhoea-predominant and post-infectious subtypes.^109^ Additionally, sugar-induced intestinal permeability increased blood endotoxin concentration,^22^^,^^43^^,^^56^ which can trigger inflammatory signalling and metabolic dysfunction, including weight gain, insulin resistance, and steatosis.^110^ Additionally, a male C57BL/6N mouse study proposed a glucagon-like peptide 2 receptor-dependent mechanism where excessive fructose consumption can exaggerate gut glucose absorption via upregulation of glucose transporters and an enlarged gut surface, which might further contribute to postprandial glucose dysregulation and liver fat accumulation.^111^

#### **Short-chain fatty acids (SCFAs)**

SCFAs play an important role in maintaining gut health by enhancing colonisation resistance against pathogens, modulating gut physiology (e.g., gut motility and transit time, mucous layer, and epithelial cell barrier), and immune regulation.^112^ A few studies found that fructose and sucrose intake can lower colonic SCFAs among rats and mice (Table 1),^26^^,^^54^ although most studies found no significant changes,^31^^,^^35^^,^^36^^,^^47^^,^^49^^,^^61^ while many gut SCFA-producing bacteria^112^ (e.g., *Clostridium*, *Eubacteriaceae*, *Ruminococcus*, and *Lachnospira*) were depleted by added sugar intake. Beyond gut health, SCFAs exert systemic health benefits, influencing extraintestinal cancers, cardiometabolic diseases, and neurological diseases.^112^ Multiple pathways are involved, such as influencing the tumour microenvironment, modulating signalling pathways in immune and cancer cells, parasympathetic effects, enhancing satiety, G protein-coupled receptors (GPCRs), histone deacetylase (HDAC) inhibition, and neurohumoral and immune pathways.^112^

#### **Bile acid metabolism**

Gut microbiota metabolises host bile acids through bile acid deconjugation and 7α-dehydroxylation, generating metabolites that signal through FXR-FGF19 and Takeda G-protein-receptor 5 pathways to regulate lipid and glucose metabolism.^113^^,^^114^ A human population study^44^ found that higher SSB intake was associated with lower abundances of gut *Eubacterium* sp. and *Clostridium* sp., which carry bile salt hydrolases (that deconjugate primary bile acids) and 7α-dehydroxylases.^113^ Less abundant gut *Eubacterium* sp. and *Clostridium* sp. were further associated with higher serum levels of ursodeoxycholic acid and glycoursodeoxycholic acid, and these two bile acids were positively associated with SSB intake, unfavourable glycemic traits, and higher diabetes risks.^44^

#### **Lipid metabolism**

Fructose intake is a major dietary risk factor for metabolic dysfunction-associated steatotic liver disease (MASLD), largely due to its potent lipogenic effects in the liver. Mechanisms include activation of ketohexokinase, unregulated accumulation of triose-phosphate intermediates fuelling de novo lipogenesis,^115^ upregulation of transcriptional regulators such as carbohydrate-responsive element-binding protein (ChREBP) and sterol regulatory element-binding protein-1c (SREBP-1c),^115^ and gut microbiota-related bile acid metabolism.^113^ Moreover, recent evidence highlights another gut microbiota-mediated pathway, whereby excess fructose alters intestinal microbial composition and function, increasing acetate production.^116^ Circulating acetate serves as an additional substrate for hepatic lipogenesis, further exacerbating liver fat accumulation.^115^

#### **Amino acid metabolism**

Gut microbiota also regulates amino acid metabolism, linking added sugar intake to cardiometabolic health. SSB-related depletion of gut *Eubacterium* sp. and *Clostridium* sp.^44^ can also catabolize aromatic amino acids (e.g., tryptophan and phenylalanine),^117-119^ and the aforementioned human population study further linked these two bacteria to three serum phenylalanine derivatives (i.e., 3-phenylpropionic acid, cinnamoylglycine, and hippuric acid) and indole−3-propionic acid (a tryptophan derivative).^44^ These aromatic amino acid derivatives modulated glucagon-like peptide−1 and anti-oxidative stress,^120-122^ and were associated with lower diabetes risk.^44^ Furthermore, this study^44^ also identified several branched-chain amino acid (BCAA) derivatives (i.e., 2-hydroxyisovaleric acid, 2-hydroxy−3-methylpentanoic acid, and (±)-2-hydroxy−4-(methylthio)butanoic acid) associated with the SSB-related gut microbial features. Particularly, *Eubacterium*, *Clostridium*, and *Ruminococcus* regulate BCAA biosynthesis and degradation,^123^ and BCAA dysmetabolism has been linked to higher diabetes risk.^124^

## Future directions

The greatest challenge is to unravel the underlying mechanisms by which added sugar intake influences the gut microbiota and human health. Given the complexity of microbial community composition and function, added sugars may modulate the gut microbiota through multiple pathways. This complexity likely contributes to the inconsistency observed across previous studies. Future research should therefore focus on mechanistic investigations to clarify how added sugars alter microbial ecology and function, and, importantly, to establish causal links between sugar-associated microbial signatures and human health outcomes—an area that remains underexplored. Particularly, integrating metabolomics approaches will be essential to identify bacterial metabolites derived from sugar metabolism that may mediate or exacerbate adverse health outcomes.

Another critical direction is to account for inter-individual variability. Differences in host genetic backgrounds,^50^^,^^51^ dietary habits,^34^^,^^46^ and lifestyles^30^ may shape how added sugars influence gut microbiota. Moreover, the sugar concentration in the gut is determined not only by intake levels but also by host absorptive capacity and microbial metabolic activities, which can influence the gut microbial responses to added sugars.^80^ Current animal evidence is also limited by the predominant use of male animals, despite female mice being more susceptible to liver steatosis.^125^ Inclusion of both sexes will provide a more comprehensive understanding of sugar-microbiome-host interactions. Furthermore, investigations into food composition and the form in which sugars are ingested (e.g., liquid vs. solid) are needed, as these factors may significantly alter microbial responses.^44^^,^^48^

Life-course perspective is another important but underexplored direction. Animal studies have shown that fructose intervention for both mothers and offspring leads to more pronounced alterations in gut microbiota in offspring,^63^ and that maternal fructose consumption can impair offspring’s metabolic health and increase oxidative stress responses,^53^^,^^63^ although it remains uninvestigated whether these physiological changes are caused by fructose-related gut microbial changes. In human populations, added sugar intake during infancy,^67^ adolescence,^65^ adulthood,^28^^,^^41^^,^^44^^,^^66^ and older age^44^^,^^66^ has been associated with gut microbial shifts. However, no single study has systematically adopted a life-course approach to examine whether added sugar consumption at different developmental or aging stages results in distinct microbial signatures, or how these stage-specific microbial changes influence long-term health trajectories. Addressing these questions will be critical for identifying sensitive windows of susceptibility and for developing age-tailored dietary guidelines and interventions.

Another important consideration is intra-species functional variation, which may contribute to the inconsistent microbial responses observed across studies. As above, different *Bacteroides* species show distinct preferences for carbohydrate fermentation substrates (e.g., glucose, fructose, or polysaccharides)^77^^,^^96-98^ and differ in their ability to utilise SCFAs through cross-feeding.^77^^,^^78^ Consequently, added sugar intake may have species-specific effects on gut *Bacteroides*. Even within a single species, strains can differ substantially in their genetic capacity for carbohydrate metabolism and ecological adaptation. For example, two epidemic *Clostridium difficile* ribotypes were found to have distinct trehalose utilisation mechanisms that enhanced virulence in mice.^126^ Such strain-dependent functional diversity highlights that taxonomic abundance alone may not necessarily reflect microbial functions. Therefore, interpretations based solely on taxonomic abundance should be made cautiously, and future studies should prioritise strain-resolved and function-based approaches to elucidate how sugar exposure affects microbial activity and metabolic outputs.

Finally, future studies are still needed to test whether dietary interventions or therapeutic strategies can reverse sugar-induced microbiome alterations and improve host health outcomes. Animal studies have reported that antibiotics and faecal transplantation can reduce the abundance of gut bacteria related to high fructose intake,^127^ as well as fructose-induced markers of metabolic syndrome, inflammation, and oxidative stress.^31^^,^^56^^,^^59^^,^^127^ Moreover, a few animal studies highlighted that exercise and supplementation of acetate and an inhibitor for trimethylamine formation may reduce the health risk caused by sugar-related gut microbiota changes.^30^^,^^35^ However, whether these interventions can be translated into clinically meaningful benefits in humans remains unknown. Besides, non-nutritive sweeteners are used as replacements for added sugars to reduce health risk. However, either their long-term health effects or their gut microbial effects remain unclear.^128^ Thus, observational population studies, human intervention studies, and real-world evidence are needed.

In summary, accumulating evidence suggests that added sugar intake may reshape gut microbial communities in ways that may contribute to multiple chronic diseases. Simple sugars can directly serve as substrates for sugar-fermenting bacteria, facilitate cross-feeding interactions, and promote the growth of facultative anaerobes under inflammatory conditions, while simultaneously suppressing strict anaerobes and SCFA-producing taxa. These microbial alterations may impair gut barrier integrity, increase inflammation, and disrupt bile acid, lipid, and amino acid metabolism, ultimately linking added sugar intake to metabolic, cardiovascular, hepatic, and gastrointestinal diseases. However, inconsistencies across studies highlight the complexity of sugar-microbiome-host interactions. Future research integrating mechanistic, multi-omics, and interventional approaches is critical to clarify causal pathways and to identify potential targets for disease prevention and treatment.
